# The Association of Walking Ability with Oral Function and Masticatory Behaviors in Community-Dwelling Older People: A Cross-Sectional Study

**DOI:** 10.3390/geriatrics9050131

**Published:** 2024-10-09

**Authors:** Takako Ujihashi, Kazuhiro Hori, Hiromi Izuno, Masayo Fukuda, Misao Sawada, Shogo Yoshimura, Shoko Hori, Fumuko Uehara, Hinako Takano, Takahiro Ono

**Affiliations:** 1 Division of Comprehensive Prosthodontics, Faculty of Dentistry, Graduate School of Medical and Dental Sciences, Niigata University, Niigata 951-8514, Japan; t-ujihashi@kobe-tokiwa.ac.jp (T.U.); syoshimura@dent.niigata-u.ac.jp (S.Y.); horisho@dent.niigata-u.ac.jp (S.H.); u-235@dent.niigata-u.ac.jp (F.U.); tkn175haru121@dent.niigata-u.ac.jp (H.T.); 2 Department of Oral Health Science, Faculty of Health Sciences, Kobe Tokiwa University, Kobe 653-0838, Japan; m-fukuda@kobe-tokiwa.ac.jp (M.F.); m-sawada@kobe-tokiwa.ac.jp (M.S.); 3 Department of Oral Health Sciences, Faculty of Nursing and Health Care, BAIKA Women’s University, Ibaraki 567-8578, Japan; kerama.blue.0123@gmail.com; 4 Department of Gerodontology, Osaka Dental College, Osaka 540-0008, Japan; ono-t@cc.osaka-dent.ac.jp

**Keywords:** walking capacity, masticatory behaviors, tongue movement, oral function, community-dwelling older people

## Abstract

**Background/Objectives**: An association between oral function and physical fitness, including walking capacity, has been reported. However, the association between masticatory behaviors and walking ability—both of which represent patterns of movement in daily life—has not been clarified. This study aimed to investigate the association between masticatory behaviors, oral function, and walking capacity in older people. **Methods**: One hundred community-dwelling older people (31 men, 69 women, mean age 75.7 ± 6.3 years) were selected to participate in this study. Age, sex, masticatory behaviors, oral functions (tongue pressure, tongue–lips motor function, occlusal force, and masticatory performance), and walking capacity were assessed. Masticatory behaviors were assessed during the consumption of one rice ball (100 g) using a wearable chewing counter, and the number of chews, chewing rate, the number of chews per bite, and meal time were recorded. Walking capacity was assessed using the timed up and go test (TUG). Spearman’s rank correlation coefficients were calculated to assess the strength and direction of the association. **Results**: Moderate negative correlations were observed between TUG time and tongue pressure and between TUG time and tongue–lips motor function (/ta/ and /ka/) (rs = −0.33, −0.21, −0.28, respectively). In addition, moderate negative correlations between TUG time and chewing rate (rs = −0.22) and between TUG time and meal time (rs = 0.33) were observed, suggesting that lower walking capacity was associated with slower chewing rate and longer meal times. **Conclusions**: In community-dwelling older people, declines in mastication speed and dexterity and tongue strength are associated with lower walking capacity.

## 1. Introduction

Major causes of older people requiring nursing care include dementia, cerebrovascular disorders (e.g., stroke), frailty, fractures, and falls [1]. In particular, it is reported that a decline in walking capacity is associated with a decline in ADL (activity of daily living) levels, perhaps as a result of a reduced desire to go out and an increased tendency to withdraw [2]. Therefore, walking is an important function for independent older people to lead an unrestricted daily life. However, slower walking speed, decreased stride length, and increased variation in pace have been observed in older people [3].

Maintaining oral function is also important for older people to live independent daily lives. Oral health affects whole-body health [4,5]. Chewing ability impacts quality of life (QOL) and health, and excellent mastication function expands food choices and eating habits and adds enjoyment to meals [6,7]. Oral health is also important to maintain a sense of well-being in daily life [8]. The number of functional teeth is associated with nutritional status [9], and maintaining or improving oral function is essential to maintain feeding function and good overall health [10].

Previous studies have reported associations between whole-body muscle weakness and oral cavities [11], occlusal support and trunk balance [12], occlusal force and falling [13], the number of teeth and walking speed [14], and occlusion and walking speed [15]. It has also been reported that masticatory function is related to prefrailty or progression to frailty [5]. Therefore, preventing tooth loss and maintaining good occlusal force may reduce the risk of falling and help to maintain physical function in daily life. Additionally, the prevalence of parafunction, including bruxism, increases with age [16]. An association between occlusion and posture has been reported [17], and parafunction might affect walking. Improving oral function is an important factor in maintaining and improving the QOL of older people [18]. However, Hatayama et al. [19] reported that a compensatory increase in the number of chews was not observed in older people with oral hypofunction, suggesting that digestion and nutrition might be affected. This means that it is necessary not only to maintain and improve oral and masticatory function, but also to modulate masticatory behavior and habits according to an individual’s state of oral and masticatory function.

A common feature of both walking and chewing is that they are patterned movements. The walking movement repeats rhythmically, with the left and right feet alternating at the same tempo, continuing automatically without conscious attention. During mastication, the lower jaw repeatedly opens and closes the mouth in a constant, unconscious rhythm. It is thought that the central pattern generator (CPG) [20] controls the rhythmic movements of both walking [21] and chewing [22].

To stably chew or walk, muscle strength and fine movement are required. In addition, chewing and walking are important for independent older people living in the community. However, the association between the two functions has not yet been studied. Therefore, we hypothesized that there might be an association between chewing and walking and related behaviors. The purpose of this study was to investigate the association between oral function, chewing behavior, and walking capacity in older people living independently. The null hypothesis of this study is that there is no association between chewing rate and walking speed.

## 2. Materials and Methods

### 2.1. Participants

This study design was cross-sectional. The participants were 100 community-dwelling, independent people aged 65 years or older who participated in senior health classes. Recruitment took place at a health course, and the survey and data collection were conducted at the health course venue from November 2019 to November 2022. Participants excluded from the study were those with a history of cerebrovascular disease, dementia, neuromuscular disease, head and neck tumors, or bone and joint disease. Moreover, participants were also excluded if they were unable to chew satisfactorily because of tooth pain or tooth mobility due to severe periodontitis when investigation; this exclusion was based on the subjective complaints of the participants themselves.

Based on the results of our preliminary experiments on the association between tongue pressure and TUG time [23], the estimated effect size was calculated to be 0.277. We decided to calculate a correlation coefficient using Spearman’s rank correlation coefficient, and the significance level was set at 0.05. A power of 80% was assumed for the two-tailed test. The sample size was calculated as 97 participants (G*Power 3.1.9.7, Heinrich-Heine-Universität Düsseldorf) [24].

The study purpose and methods were fully explained to the participants in advance, and their informed consent was obtained. This study was conducted with the approval of the Niigata University Ethics Committee (approval number: 2017-0230).

### 2.2. Data Collection

Basic information, walking capacity, and masticatory behaviors were assessed. For data collection, denture wearers were evaluated with their dentures in place.

#### 2.2.1. Basic Information

The height, weight, and body mass index of all participants were measured. A questionnaire about medical history, medication, smoking, fitness habits, coughing while eating, sputum production, and usage of removable dentures was completed, and the dentist (K.H.) checked the number of remaining teeth.

#### 2.2.2. Walking Capacity

Walking capacity was assessed using the timed up and go test (TUG). The TUG test was originally developed as a clinical measure of balance in older people [25]. The TUG test measures the time it takes a participant to stand up from an armchair, walk a distance of three meters, turn, walk back to the chair, and sit down. The TUG test was performed twice, and the faster time was used as the score.

#### 2.2.3. Masticatory Behaviors

Masticatory behaviors were measured using a device to count the number of chews (bitescan^®^, Sharp Co., Ltd., Sakai, Japan) [26]. The device is designed to be worn on the right auricle with an ear hook. The ear hook is available in three sizes (S, M, L), and the size that best fit the auricle of the participant was selected to enable the built-in sensor to sense the measurement site behind the auricle. For measurement, a Bluetooth connection with a smartphone (SHM05, Sharp Co., Ltd., Sakai, Japan) was confirmed and calibration was carried out. For the assessment of masticatory behaviors, the participant was asked to eat a 100 g rice ball (seaweed-rolled rice ball, Marusan Co., Ltd., Higashi-Osaka, Japan). No special eating instructions were given. The test was carried out at least 2 h after meals.

As parameters of masticatory behaviors, the number of chews, the number of chews per bite, chewing rate, and total meal time were measured. Masticatory behaviors were defined as follows:

- Number of chews: the total number of chewing cycles over the time taken to eat one rice ball.
- Number of chews per bite: the mean number of chews per bite, with a bite defined as a food intake action.
- Chewing rate (number/min): the number of chews per minute, calculated by dividing the total number of chews by the total meal time.
- Meal time (s): the time taken to eat one rice ball.

#### 2.2.4. Oral Function

Oral function assessment included measurement of maximal tongue pressure, tongue–lips motor function, occlusal force, and masticatory performance.

##### Maximal Tongue Pressure

Maximal tongue pressure was measured using a digital probe (JMS tongue pressure measuring device TPM-02E, JMS Co., Ltd., Hiroshima, Japan) [27]. A balloon was placed against the anterior part of the palate, and the participant was instructed to voluntarily squash the balloon against the palate using the tongue with maximal force for 7 s. After the participant first practiced and then rested to avoid fatigue, measurements were carried out three times, and the mean value was calculated.

##### Tongue–Lips Motor Function

The speed and dexterity of tongue and lips movements were evaluated using oral diadochokinesis. Participants were required to pronounce each of the syllables /pa/, /ta/, and /ka/ for 5 s, and the number of pronunciations per second of each syllable was measured using an automatic measuring device (Kenko-kun Handy, Takei Scientific Instruments Co., Ltd., Niigata, Japan) [28].

##### Occlusal Force

Occlusal force was analyzed using a pressure-sensitive sheet (Dental Prescale II, GC Co., Ltd., Tokyo, Japan) [29] and an analysis device (Bite Force Analyzer, GC Co., Ltd., Tokyo, Japan). The pressure-sensitive sheet was placed between the upper and lower dentition, and the participant was instructed to clench their teeth for 3 s in the maximal intercuspal position.

##### Masticatory Performance

Masticatory performance was measured using a test gummy jelly (5.50 ± 0.05 g, UHA Mikakuto Co., Ltd., Osaka, Japan) [30]. The participant was instructed to chew the gummy jelly 30 times and then spit it out into a gauze. The comminuted gummy jelly was then placed in a standardized box (inner dimensions 140 mm × 95 mm × 36 mm) with black markers (7 mm × 7 mm—width 88 mm, length 133 mm). The increase in surface area of the comminuted gummy jelly was calculated using an imaging method [31].

### 2.3. Statistical Analysis

Correlations between walking capacity, masticatory behaviors, and oral functions were evaluated using Spearman’s rank correlation coefficient. SPSS version 29.0J for Windows (IBM Japan, Tokyo, Japan) was used for statistical analysis. Statistical significance was set at *p* < 0.05.

## 3. Results

### 3.1. Basic Information

The total number of study participants was 100 (31 men, 69 women, average age 75.7 ± 6.3 years). Participant characteristics are shown in Table 1. The median TUG time was 7.36 s (interquartile range 6.22–8.46 s). According to Shumway-Cook et al. [32], the cutoff value for TUG time that indicates a high risk of falling is 13.5 s. Only one participant exceeded 13.5 s, with a TUG score of 13.57 s. Therefore, few participants had any obvious walking disorders.

### 3.2. The Association between Masticatory Behaviors, Age, and Walking Capacity

Table 2 shows the results of the correlation analysis between masticatory behaviors and walking capacity. A moderate negative correlation between chewing rate and TUG time (rs = −0.217, *p* = 0.030) was observed, as was a moderate positive correlation between total meal time and TUG time (rs = 0.325, *p* = 0.001). Significant correlations were not found between TUG time and the number of chews or the number of chews per bite.

Table 2 shows the results of the correlation analysis between masticatory behaviors and age. Significant correlations were not found between age and masticatory behaviors.

### 3.3. The Association between Oral Function, Age, and Walking Capacity

Table 3 shows the results of the correlation analysis between oral function and walking capacity. Moderate negative correlations were observed between TUG time and maximum tongue pressure (rs = −0.325, *p* = 0.001), oral diadochokinesis /ta/ (rs = −0.213, *p* = 0.034), and oral diadochokinesis /ka/ (rs = −0.281, *p* = 0.005). Significant correlations were not found between TUG time and oral diadochokinesis /pa/, occlusal force, or masticatory performance.

Table 3 shows the results of the correlation analysis between oral function and age. Moderate negative correlations were observed between age and maximum tongue pressure (rs = −0.321, *p* = 0.001), oral diadochokinesis /ta/ (rs = −0.217, *p* = 0.030), and oral diadochokinesis /ka/ (rs = −0.253, *p* = 0.011). Significant correlations were not found between age and oral diadochokinesis /pa/, occlusal force, or masticatory performance.

A relatively strong positive correlation was observed between age and walking capacity (rs = 0.548, *p* < 0.001).

## 4. Discussion

This study is the first to investigate the association between masticatory behaviors, oral function, and walking capacity. We obtained evidence of associations between TUG time and masticatory behaviors (chewing rate and total meal time) and between TUG time and oral function (tongue pressure and oral diadochokinesis /ta/, /ka/). The results of this study may indicate that physical frailty has an association not only with oral health but also with masticatory habits, which is one type of eating habit.

This study found evidence of an association between slower TUG time and lower tongue pressure, confirming previous research conducted by Izuno et al. [23]. Additional research has shown that muscle fibers in the tongue atrophy or disappear with age [33]. Associations have also been found between tongue pressure, grip strength, and jumping strength [34,35]. Therefore, it is likely that age-related changes in tongue function, including mastication, swallowing, and articulation, are also related to changes in perioral organs and tissues. Okada et al. [36] have also reported an association between protein intake and walking speed. Protein is needed to increase muscle mass and strengthen muscle. However, it has been reported that older people with poor masticatory function consume less protein [37]. It is thought that these factors may lead to decreased muscle mass throughout the body, including lower limb strength, which may result in slower walking speeds. The results of this study also suggest that there may be an association between a decline in walking capacity caused by whole-body muscle weakness and a decline in tongue muscle strength, which plays an important role during mastication.

We found a negative association between oral diadochokinesis for /ta/ and /ka/ and TUG time. Previous research has shown that physical dexterity [38] and agility [39] decrease with age. The TUG test is a test that includes standing up, changing direction, and sitting down, in addition to walking in a straight line. Therefore, it has been recognized that prolonged TUG times result not only from whole-body muscle weakness but also from a sense of imbalance, ease of falling, a lack of agility [32], and functional immobility [40].

Oral diadochokinesis with the syllables /pa/, /ta/, and /ka/ was used to evaluate the motor function of the tongue and lips. In general, /pa/ evaluates the motor dexterity of the lips, /ta/ the anterior tongue, and /ka/ the posterior tongue. In a previous study by Murotani et al. [41], no association was found between occlusal force and walking speed, but a significant correlation was found between oral diadochokinesis /pa/ and swallowing function and walking speed. This suggests that complex functions such as oral diadochokinesis and swallowing function are closely related to walking speed. Our past research [23] also found an association between oral diadochokinesis (/pa/, /ta/, and /ka/) and TUG time, and an association between tongue lateral movement (evaluating tongue dexterity) and TUG time. Therefore, it is thought that the dexterity and agility of the tongue are related to functional mobility. These results suggest that decreased speed of movement of the lower limbs may be related to slower repetitive movements of the oral organs, that is, the dexterity of the tongue.

A moderate negative correlation was observed between chewing rate and TUG time, and a moderate positive correlation was observed between meal time and TUG time. Therefore, the null hypothesis we set was rejected. These results suggest that older people who walk more slowly also chew at a slower rate, prolonging food ingestion time. Chewing movements are classified as semi-automatic movements. Breathing and walking are also classified as semi-automatic movements [42] and are controlled by the CPG [43,44]. The start and stop of these behaviors can be controlled freely, but the movement itself is periodic and follows a rhythm. Such rhythmic movements are reported to respond to environmental changes [45].

It is assumed that the cerebral cortex integrates the masticatory, swallowing, and respiratory motor centers, each of which sends independent commands [21]. Signals from the midbrain gait-inducing area are thought to activate the CPG and the muscle tone facilitator system in the brainstem and spinal cord to induce walking [46]. By contrast, it has been suggested that the masticatory CPG, which is involved in the control of masticatory movements, does not necessarily require input from the higher brain and exists in an area adjacent to the central pathway for sensory information from the oral cavity. The locations of the CPGs controlling walking and mastication are thought to be different, and the association between the two CPGs is not known. However, our previous research found that TUG time was related to oral function [23] and to chewing rate [19].

A negative association was found between age and oral function, though no significant association between age and masticatory behaviors was found. Hatanaka et al. reported that although there was large variability in oral function, negative correlations were found between age and tongue pressure and between age and oral diadochokinesis [47]. As muscle strength declines with age, tongue movement and strength are expected to decrease as well. In fact, this study showed that age exhibited a relatively strong positive correlation with walking speed, suggesting that leg muscle strength decreases with age.

For older people to live independent daily lives for as long as possible, it is important to monitor small declines in physical condition and detect them as early as possible. Mihara et al. [48] suggested that grip strength not only indicates general muscle strength but may also predict general health status. The authors suggested that the provision of dental treatment to older people provides an opportunity to test a patient’s grip strength; in cases of lowered grip strength, there is a risk that other functions might also decline. Murotani et al. [41] have suggested an association between oral function and muscle strength (grip strength) and between oral motor function and physical function (walking speed). Therefore, oral function assessment could be useful as an alternative indicator of physical decline in older people and may be used to screen for physical frailty. In our study, no evidence of an association was found between the number of teeth and TUG time, but associations were found for chewing rate and meal time. This result is consistent with a previous finding that in a group of 80-year-old participants, occlusal support was associated with decreased walking speed and was more strongly related to motor function than the number of teeth [49]. Older people, to stabilize their posture, tend to clench their teeth, and occlusion should be associated with their posture and stability [17]. Based on these results, if the walking speed of older people decreases, it may indicate that their masticatory function is also decreasing. It is important to include foods of various firmness levels in the daily diet of older people and to encourage them to chew stably at a tempo that is comfortable according to their own oral and masticatory function.

In this study, we investigated the association between oral function, masticatory behaviors, and walking capacity in community-dwelling, independent older people. However, there are several limitations to this study. It was cross-sectional in design, and therefore, it was not possible to establish causality. In addition, this study included only independent older people living in the community and did not include participants with lower limb diseases such as arthritis that interfere with walking ability. Factors related to walking speed include exercise habits, the frequency of going outside, experiences of falling, muscle mass, toe strength, balance ability, the range of motion of joints, and eyesight. The analyses herein were limited to simple, univariate correlations. Multiple factors are related to walking, and multivariate analyses will be necessary in the future to reduce confounding.

TUG time was used as a proxy for walking capacity in this study. However, the TUG test measures not only walking speed but also factors such as balance, ease of falling, and agility, including standing up, changing direction, and sitting down. Other common measures used to evaluate walking include the 10 m walk measurement [50] and 6 min walk test [51]. However, in this study, participants were older, and the burden on their bodies was taken into consideration. In addition, the measurement area available was limited in size. Chewing rate was calculated based on the total meal time and number of chews. It may have been useful to also consider chewing rate stability.

The mean number of teeth of the participants in this study was 21.7. This number of teeth was higher than the average of the Japanese older population. In addition, the oral interest of participants might be slightly higher, as indicated by spontaneous participation in health classes.

In the future, we intend to conduct large-scale, longitudinal surveys to further clarify the effects of oral function and chewing behavior on walking capacity.

## 5. Conclusions

Lower masticatory speed and reduced tongue strength and dexterity are associated with decreased walking capacity in community-dwelling, independent older people.

## Figures and Tables

**Table 1:** Basic information.

	Median	Interquartile Range	
		Lower	Higher
Height (cm)	154.3	149.1	162.0
Weight (kg)	55.5	49.4	63.3
BMI ^a^	23.1	21.3	25.7
Time of TUG ^b^ (s)	7.36	6.22	8.46
Masticatory behaviors			
Number of chews	230.5	168.3	280.8
Number of chews per bite	25.4	18.2	42.9
Chewing rate (/min)	78.0	68.0	84.5
Meal time (s)	195	151	257
Number of teeth remaining	24	20	27
Oral function			
Tongue pressure (kPa)	30.6	25.9	37.2
Diadochokinesis /pa/ (/s)	6.4	6.0	6.8
/ta/ (/s)	6.3	5.8	6.8
/ka/ (/s)	5.8	5.4	6.4
Occlusal force (N)	607.2	371.5	944.1
Masticatory performance (mm^2^)	3500.7	2070.8	4334.2

^a^ BMI: body mass index. ^b^ TUG: timed up and go test.

**Table 2:** Correlations between masticatory behaviors, age, and time of TUG ^a^.

	Time of TUG ^a^				Age			
	rs ^b^	95% CI ^c^		*p*	rs ^b^	95% CI ^c^		*p*
		Lower	Higher			Lower	Higher	
Masticatory behaviors								
Number of chews	0.157	−0.046	0.348	0.118	0.079	−0.126	0.276	0.437
Number of chews per bite	−0.049	−0.249	0.155	0.628	−0.006	−0.208	0.196	0.953
Chewing rate (/min)	−0.217	−0.401	−0.016	0.030	−0.076	−0.274	0.128	0.453
Meal time (s)	0.325	0.132	0.495	0.001	0.177	−0.026	0.366	0.078

^a^ TUG: timed up and go test. ^b^ Spearman’s rank correlation coefficient. ^c^ CI: confidence interval.

**Table 3:** Correlation between oral function, age, and time of TUG ^a^.

	Time of TUG ^a^				Age			
	rs ^b^	95% CI ^c^		*p*	rs ^b^	95% CI ^c^		*p*
		Lower	Higher			Lower	Higher	
Oral function								
Tongue pressure (kPa)	−0.325	−0.494	−0.131	0.001	−0.321	−0.491	−0.127	0.001
Diadochokinesis /pa/ (/s)	−0.109	−0.304	0.096	0.282	−0.144	−0.336	0.060	0.152
/ta/ (/s)	−0.213	−0.398	−0.011	0.034	−0.217	−0.401	−0.015	0.030
/ka/ (/s)	−0.281	−0.457	−0.084	0.005	−0.253	−0.433	−0.054	0.011
Occlusal force (N)	0.035	−0.170	0.237	0.732	−0.069	−0.269	0.138	0.503
Masticatory performance (mm^2^)	−0.139	0.132	0.495	0.196	−0.151	−0.355	0.067	0.161

^a^ TUG: timed up and go test. ^b^ Spearman’s rank correlation coefficient. ^c^ CI: confidence interval.

## Data Availability

The data presented in this study are available on request from the corresponding author. The data are not publicly available because of ethical restrictions.

## References

[1] Ministry of Health L.a.W. Summary Report of Comprehensive Survey of Living Conditions 2019. https://www.mhlw.go.jp/english/database/db-hss/dl/report_gaikyo_2019.pdf.

[2] Mendes de Leon C.F., Seeman T.E., Baker D.I., Richardson E.D., Tinetti M.E. (1996). Self-efficacy, physical decline, and change in functioning in community-living elders: A prospective study. J. Gerontol. B Psychol. Sci. Soc. Sci..

[3] Menz H.B., Lord S.R., Fitzpatrick R.C. (2003). Age-related differences in walking stability. Age Ageing.

[4] Yoshida M., Hiraoka A., Takeda C., Mori T., Maruyama M., Yoshikawa M., Tsuga K. (2022). Oral hypofunction and its relation to frailty and sarcopenia in community-dwelling older people. Gerodontology.

[5] Horibe Y., Ueda T., Watanabe Y., Motokawa K., Edahiro A., Hirano H., Shirobe M., Ogami K., Kawai H., Obuchi S. (2018). A 2-year longitudinal study of the relationship between masticatory function and progression to frailty or pre-frailty among community-dwelling Japanese aged 65 and older. J. Oral Rehabil..

[6] Ikebe K., Hazeyama T., Morii K., Matsuda K., Maeda Y., Nokubi T. (2007). Impact of masticatory performance on oral health-related quality of life for elderly Japanese. Int. J. Prosthodont..

[7] McGrath C., Bedi R. (1999). The importance of oral health to older people’s quality of life. Gerodontology.

[8] Brennan D.S., Spencer A.J., Roberts-Thomson K.F. (2008). Tooth loss, chewing ability and quality of life. Qual. Life Res..

[9] Toniazzo M.P., Amorim P.S., Muniz F., Weidlich P. (2018). Relationship of nutritional status and oral health in elderly: Systematic review with meta-analysis. Clin. Nutr..

[10] Kossioni A.E. (2018). The Association of Poor Oral Health Parameters with Malnutrition in Older Adults: A Review Considering the Potential Implications for Cognitive Impairment. Nutrients.

[11] Yamaga T., Yoshihara A., Ando Y., Yoshitake Y., Kimura Y., Shimada M., Nishimuta M., Miyazaki H. (2002). Relationship between dental occlusion and physical fitness in an elderly population. J. Gerontol. A Biol. Sci. Med. Sci..

[12] Julia-Sanchez S., Alvarez-Herms J., Burtscher M. (2019). Dental occlusion and body balance: A question of environmental constraints?. J. Oral Rehabil..

[13] Eto M., Miyauchi S. (2018). Relationship between occlusal force and falls among community-dwelling elderly in Japan: A cross-sectional correlative study. BMC Geriatr..

[14] Welmer A.K., Rizzuto D., Parker M.G., Xu W. (2017). Impact of tooth loss on walking speed decline over time in older adults: A population-based cohort study. Aging Clin. Exp. Res..

[15] Inui A., Takahashi I., Sawada K., Naoki A., Oyama T., Tamura Y., Osanai T., Satake A., Nakaji S., Kobayashi W. (2016). Teeth and physical fitness in a community-dwelling 40 to 79-year-old Japanese population. Clin. Interv. Aging.

[16] Zielinski G., Pajak A., Wojcicki M. (2024). Global Prevalence of Sleep Bruxism and Awake Bruxism in Pediatric and Adult Populations: A Systematic Review and Meta-Analysis. J. Clin. Med..

[17] Nowak M., Golec J., Wieczorek A., Golec P. (2023). Is There a Correlation between Dental Occlusion, Postural Stability and Selected Gait Parameters in Adults?. Int. J. Environ. Res. Public Health.

[18] Okuyama N., Yamaga T., Yoshihara A., Nohno K., Yoshitake Y., Kimura Y., Shimada M., Nakagawa N., Nishimuta M., Ohashi M. (2011). Influence of dental occlusion on physical fitness decline in a healthy Japanese elderly population. Arch. Gerontol. Geriatr..

[19] Hatayama C., Hori K., Izuno H., Fukuda M., Sawada M., Ujihashi T., Yoshimura S., Hori S., Togawa H., Uehara F. (2022). Features of Masticatory Behaviors in Older Adults with Oral Hypofunction: A Cross-Sectional Study. J. Clin. Med..

[20] Tsuboi A., Kolta A., Chen C.C., Lund J.P. (2003). Neurons of the trigeminal main sensory nucleus participate in the generation of rhythmic motor patterns. Eur. J. Neurosci..

[21] Kiehn O. (2006). Locomotor circuits in the mammalian spinal cord. Annu. Rev. Neurosci..

[22] Morquette P., Lavoie R., Fhima M.D., Lamoureux X., Verdier D., Kolta A. (2012). Generation of the masticatory central pattern and its modulation by sensory feedback. Prog. Neurobiol..

[23] Izuno H., Hori K., Sawada M., Fukuda M., Hatayama C., Ito K., Nomura Y., Inoue M. (2016). Physical fitness and oral function in community-dwelling older people: A pilot study. Gerodontology.

[24] Faul F., Erdfelder E., Lang A.G., Buchner A. (2007). G*Power 3: A flexible statistical power analysis program for the social, behavioral, and biomedical sciences. Behav. Res. Methods.

[25] Mathias S., Nayak U.S., Isaacs B. (1986). Balance in elderly patients: The “get-up and go” test. Arch. Phys. Med. Rehabil..

[26] Hori K., Uehara F., Yamaga Y., Yoshimura S., Okawa J., Tanimura M., Ono T. (2021). Reliability of a novel wearable device to measure chewing frequency. J. Prosthodont. Res..

[27] Hayashi R., Tsuga K., Hosokawa R., Yoshida M., Sato Y., Akagawa Y. (2002). A novel handy probe for tongue pressure measurement. Int. J. Prosthodont..

[28] Yamada A., Kanazawa M., Komagamine Y., Minakuchi S. (2015). Association between tongue and lip functions and masticatory performance in young dentate adults. J. Oral Rehabil..

[29] Suzuki T., Kumagai H., Watanabe T., Uchida T., Nagao M. (1997). Evaluation of complete denture occlusal contacts using pressure-sensitive sheets. Int. J. Prosthodont..

[30] Nokubi T., Yoshimuta Y., Nokubi F., Yasui S., Kusunoki C., Ono T., Maeda Y., Yokota K. (2013). Validity and reliability of a visual scoring method for masticatory ability using test gummy jelly. Gerodontology.

[31] Salazar S., Hori K., Uehara F., Okawa J., Shibata A., Higashimori M., Nokubi T., Ono T. (2020). Masticatory performance analysis using photographic image of gummy jelly. J. Prosthodont. Res..

[32] Shumway-Cook A., Brauer S., Woollacott M. (2000). Predicting the probability for falls in community-dwelling older adults using the Timed Up & Go Test. Phys. Ther..

[33] Newton J.P., Yemm R., Abel R.W., Menhinick S. (1993). Changes in human jaw muscles with age and dental state. Gerodontology.

[34] Maeda K., Akagi J. (2015). Decreased tongue pressure is associated with sarcopenia and sarcopenic dysphagia in the elderly. Dysphagia.

[35] Buehring B., Hind J., Fidler E., Krueger D., Binkley N., Robbins J. (2013). Tongue strength is associated with jumping mechanography performance and handgrip strength but not with classic functional tests in older adults. J. Am. Geriatr. Soc..

[36] Okada T., Ikebe K., Kagawa R., Inomata C., Takeshita H., Gondo Y., Ishioka Y., Okubo H., Kamide K., Masui Y. (2015). Lower Protein Intake Mediates Association Between Lower Occlusal Force and Slower Walking Speed: From the Septuagenarians, Octogenarians, Nonagenarians Investigation with Centenarians Study. J. Am. Geriatr. Soc..

[37] Iwasaki M., Yoshihara A., Ogawa H., Sato M., Muramatsu K., Watanabe R., Ansai T., Miyazaki H. (2016). Longitudinal association of dentition status with dietary intake in Japanese adults aged 75 to 80 years. J. Oral Rehabil..

[38] Smith C.D., Umberger G.H., Manning E.L., Slevin J.T., Wekstein D.R., Schmitt F.A., Markesbery W.R., Zhang Z., Gerhardt G.A., Kryscio R.J. (1999). Critical decline in fine motor hand movements in human aging. Neurology.

[39] Pedrero-Chamizo R., Gomez-Cabello A., Melendez A., Vila-Maldonado S., Espino L., Gusi N., Villa G., Casajus J.A., Gonzalez-Gross M., Ara I. (2015). Higher levels of physical fitness are associated with a reduced risk of suffering sarcopenic obesity and better perceived health among the elderly: The EXERNET multi-center study. J. Nutr. Health Aging.

[40] Podsiadlo D., Richardson S. (1991). The timed “Up & Go”: A test of basic functional mobility for frail elderly persons. J. Am. Geriatr. Soc..

[41] Murotani Y., Hatta K., Takahashi T., Gondo Y., Kamide K., Kabayama M., Masui Y., Ishizaki T., Matsuda K.I., Mihara Y. (2021). Oral Functions Are Associated with Muscle Strength and Physical Performance in Old-Old Japanese. Int. J. Environ. Res. Public Health.

[42] Feldman J.L., Mitchell G.S., Nattie E.E. (2003). Breathing: Rhythmicity, plasticity, chemosensitivity. Annu. Rev. Neurosci..

[43] McCrea D.A., Rybak I.A. (2008). Organization of mammalian locomotor rhythm and pattern generation. Brain Res. Rev..

[44] Rossignol S., Dubuc R., Gossard J.P. (2006). Dynamic sensorimotor interactions in locomotion. Physiol. Rev..

[45] Bizzi E., Tresch M.C., Saltiel P., d’Avella A. (2000). New perspectives on spinal motor systems. Nat. Rev. Neurosci..

[46] MacKay-Lyons M. (2002). Central pattern generation of locomotion: A review of the evidence. Phys. Ther..

[47] Hatanaka Y., Furuya J., Sato Y., Uchida Y., Shichita T., Kitagawa N., Osawa T. (2021). Associations between Oral Hypofunction Tests, Age, and Sex. Int. J. Environ. Res. Public Health.

[48] Mihara Y., Matsuda K.I., Ikebe K., Hatta K., Fukutake M., Enoki K., Ogawa T., Takeshita H., Inomata C., Gondo Y. (2018). Association of handgrip strength with various oral functions in 82- to 84-year-old community-dwelling Japanese. Gerodontology.

[49] Hatta K., Ikebe K., Mihara Y., Gondo Y., Kamide K., Masui Y., Sugimoto K., Matsuda K.I., Fukutake M., Kabayama M. (2019). Lack of posterior occlusal support predicts the reduction in walking speed in 80-year-old Japanese adults: A 3-year prospective cohort study with propensity score analysis by the SONIC Study Group. Gerodontology.

[50] Peters D.M., Fritz S.L., Krotish D.E. (2013). Assessing the reliability and validity of a shorter walk test compared with the 10-Meter Walk Test for measurements of gait speed in healthy, older adults. J. Geriatr. Phys. Ther..

[51] Enright P.L. (2003). The six-minute walk test. Respir. Care.

